# The cGAS-STING pathway in acute myocardial infarction: Biomarker discovery and mechanistic insights

**DOI:** 10.1371/journal.pone.0355694

**Published:** 2026-09-18

**Authors:** Juying Li, Hongkui Chen, Yihao Huang, Yupeng Zhi, Chun Chen, Yansong Guo

**Affiliations:** 1 Shengli Clinical Medical College of Fujian Medical University, Fuzhou, Fujian, China; 2 Department of Cardiology, The First People’s Hospital of Yibin, Yibin, Sichuan, China; 3 School of Pharmacy, Fujian Medical University, Fuzhou, Fujian, China; Second Xiangya Hospital, CHINA

## Abstract

Acute Myocardial infarction (AMI) continues to be a severe deadly disease with a high case-fatality rate and great psychological and economic costs. Despite the development of new therapies, early diagnosis and treatment optimization still are problematical. Here we applied the integrated bioinformatics strategy to screen potential new diagnosis biomarkers for AMI based on the inflammation-related cGAS-STING pathway. By integrating differentially expressed genes (DEGs), weighted gene co-expression network analysis (WGCNA), and cGAS-STING gene set from high-throughput RNA sequencing, we obtained a total of 11 candidate genes from 914 DEGs, which enriched mainly for inflammatory and immune response pathways. Further refining them in machine learning algorithms, we identified core genes with diagnostic values. Subsequent test shows the AUC values of PFKFB3 and TLR2 both were more than 0.7 and their promising diagnostic values were verified. Based on the immune infiltration analysis, expression of PFKFB3 and TLR2 were very significantly associated with a large number of immune cells, such as monocytes, neutrophils and dendritic cells. As for single cell sequencing analysis, they were mainly found expressed on macrophages. Overall, we show that PFKFB3 and TLR2 are possible biomarkers of AMI which have not been reported so far. Thus they may open a path for new diagnostics and personalized therapy. Validation in independent populations is needed.

## Introduction

Acute myocardial infarction (AMI), as a common cardiovascular disease with the highest number of global incidences and deaths, is still a major public health problem concerning human wellbeing. In recent years, cardiovascular diseases have increased over a gradually higher amount, and AMI has become one of the main public health problems caused by sudden onset, poor prognosis and tendency towards recurrence. According to large-scale epidemiological surveys, millions of people in the world lose their lives each year to various cardiovascular diseases, among them myocardial infarction (MI or AMI) is a very big part [1]. In addition to affecting human individual health, it also incurs a huge economic and social burden to the country [2]. Despite the recent improvements in medicine, the incidence and death rate of AMI cannot be effectively inhibited [3]. This is at least in part due to the complex pathophysiological mechanisms of the disease and limitations in the existing early diagnostic method [1].

Currently, a well-established clinical management framework for AMI has been developed, encompassing pharmacological treatment, interventional procedures, and rehabilitation strategies, among others [3]. However, diagnosis based on conventional clinical symptoms, electrocardiographic findings, and serum biomarkers frequently faces challenges in early detection due to limited specificity or sensitivity [4]. Meanwhile, despite undergoing standardized therapy, a number of patients continue to develop adverse events, including impaired cardiac function, arrhythmias, and disease recurrence, indicating that existing diagnostic and treatment strategies have certain constraints in optimizing long-term outcomes [5]. Therefore, there is an urgent need to elucidate the molecular mechanisms underlying the onset and development of AMI to enable earlier and more accurate diagnosis as well as timely therapeutic intervention.

With the progression of the field in molecular biology and immunology the participation of the cGAS-STING pathway (cyclic GMP-AMP synthase–stimulator of interferon genes pathway) in cardiovascular diseases is attracting growing scientific attention. cGAS-STING pathway, a central pathway for the activation of the innate immune system due to the sensing of cytosolic DNA, has already been shown to play major regulatory role in various inflammatory processes as well as in processes of tissue damage [6,7]. The dysregulated activation of the cGAS-STING pathway can cause sterile inflammation, which in turn can lead to exacerbated tissue injury and cell apoptosis. Its role in the pathogenesis of some CVDs such as myocardial ischemia reperfusion injury and atherosclerosis has recently been extensively revealed [8,9]. While previous studies have shown that this pathway has a significant relationship with cardiovascular diseases, however, the detailed molecular mechanism and downstream regulation network on initiation and progression of AMI is not completely clear yet and more detail investigation in the clinical benefit of biomarkers associated to this pathway is required.

Under these circumstances, comprehensive and integrated omics explorations of the cGAS-STING pathway-related molecules in AMI are strongly needed to explore key regulatory hubs during disease process and detect biomarkers with promising clinical use. Although these studies highlighted the functional validation of single molecules or individual signaling pathways, a global integration of multi-omics data is urgently needed to get an overall insight into the pathway and downstream regulatory network [10]. Second, since immune-inflammatory, cell death and tissue healing mechanisms also play complex roles in the developing process of myocardial infarction, basing a diagnosis or even a risk stratification strategy solely on one biomarker is inappropriate. Therefore, data-centric, multi-dimensional bioinformatics methods provide unique benefit to myocardial infarction and may enable discovery of novel potential molecular targets that may have been otherwise overlooked through traditional methods.

In this work, different from previous works, high through put RNA sequencing datasets, combined with differential expression analysis, functional enrichment, and WGCNA, were used as the multi-omics integration toolbox to systematically find those candidate genes of the cGAS-STING pathway in AMI patients. Moreover, we used the machine learning approaches to evaluate the diagnostic value of these genes, and immune infiltration analysis and molecular regulatory networks building were performed to further explain the potential mechanism of these significant genes. Through departing the scope of single-gene studies, this approach will allow the merging of multiple dimensions of data, presents an overview of the molecular landscape at the time of onset for AMI, and gives an appropriate foundation for downstream functional validation and translational research [11].

Overall, we aim to identify and test cGAS-STING pathway related biomarkers systematically in AMI, as well as investigating their potential functions in the regulation of immune-inflammatory responses. We will construct an integrated molecular network for AMI using the multimodal omics bioinformatics methodologies to identify the major genes with high power in diagnosis and more prospects for clinical application. The above aim is to provide firm theoretical basis and practical molecular tool for the early diagnosis and precise treatment of AMI to improve development of precision medicine of cardiovascular diseases.

## Materials and Methods

### Ethical statement and consent

This study was approved by the Ethics Committee of the Fujian Medical University (approval number: IACUC FJMU 2023−0210). Written informed consent was obtained from all the participants.

### Data source

The gene expression dataset GSE66360, GSE48060 and single-cell RNA sequencing (scRNA-seq) data GSE180678 were retrieved from the NCBI Gene Expression Omnibus (GEO) database [12]. The training set GSE66360 (AMI = 49, control = 50, ID: 200066360) and the validation set GSE48060 (AMI = 31, control = 21, ID: 200048060) were generated using the GPL570 platform (Affymetrix Human Genome U133 Plus 2.0 Array, [HG-U133_Plus_2]). The single-cell RNA sequencing (scRNA-seq) data utilized in this study were obtained from the GSE180678 dataset (ID:200180678). This dataset contains myocardial tissue profiled from one patient with AMI, generated on the GPL20301 sequencing platform. Genes associated with the cGAS-STING pathway were compiled from three public databases: PathCards yielded 103 genes [PMID: 40035384]; MsigDB identified 18 genes (KEGG_MEDICUS_REFERENCE_cGAS_TING_SIGNALING_PATHWAY); and GeneCards retrieved 466 genes. A union of these gene sets was constructed for subsequent analysis(S1 Table).

### Visualization of differentially expressed genes (DEGs)

DEGs in the training set were identified by comparing AMI samples to healthy controls using the limma package (version 3.62.2), with the threshold set at |log2FC| > 0.5 and an adjusted P-value < 0.05 [13]. The resulting DEGs were presented via a volcano plot and a heatmap (version 1.0.13).

### Weighted gene co-expression network analysis (WGCNA)

We used WGCNA, implemented in the corresponding R package, to obtain clusters of highly correlated genes (modules), co-expressed in association with AMI [14]. Briefly, we first used the top 5000 most variable genes from the AMI expression matrix to build the network, and chose an optimal soft-thresholding power which best approximates scale-free topology. An adjacency matrix was then computed and transformed to a topological overlap matrix (TOM). We next applied hierarchical clustering to identify a number of clusters (modules) of genes that had highly correlated expression across samples. We then computed the correlation between each module eigengene and the disease trait and selected the module with highest positive correlation (P < 0.05) to conduct follow-up analysis.

### Identification of Candidate Genes

To get the key candidate genes, the intersection among DEGs, genes in the key AMI-associated WGCNA module and genes in cGAS-STING pathway was calculated. The Venn diagram was created based on “VennDiagram” R package and the genes that overlapped among three groups of genes were gathered for subsequent analysis as the final candidate genes.

### GO and KEGG Enrichment Analysis of Candidate Genes

Functional enrichment analysis of the candidate genes was performed using the R package clusterProfiler [15]. This included Kyoto Encyclopedia of Genes and Genomes (KEGG) pathway analysis and Gene Ontology (GO) term enrichment. Terms with a p-value < 0.05 and a count > 1 were considered statistically significant.

### Construction of Protein-Protein Interaction (PPI) Networks

A PPI network for the candidate genes was constructed using the STRING database (version 12.0) [16]. Interactions with a combined confidence score > 0.40 were retrieved. The resulting network was then imported and visualized using Cytoscape (version 3.10.1) [17].

### Feature genes are screened by machine learning algorithms

A multi-stage machine learning approach was employed to refine diagnostic biomarkers from candidate hub genes [18]. First, the Boruta algorithm (R package, version 8.0.0) was used for an all-relevant feature selection, retaining only attributes confirmed as significant after Bonferroni correction (p < 0.01) [19]. Subsequently, the Random Forest (RF) package (version 4.7–1.2) and the glmnet package (version 4.1–9) for LASSO regression were applied for further feature selection [18,20]. The final signature genes were defined as the intersecting set identified by all three algorithms.

### Diagnostic power and expression levels

The diagnostic performance of the feature genes was evaluated using the pROC R package (version 1.18.5) [21]. Receiver operating characteristic (ROC) curves were generated for both the training and validation AMI datasets. Genes that consistently achieved an area under the curve (AUC) greater than 0.7 across all datasets were defined as candidate biomarkers. The candidate biomarkers underwent systematic cross-cohort validation using independent training and validation AMI datasets. Their ability to distinguish disease states was assessed by comparing expression levels between AMI and control groups using the Wilcoxon test in both cohorts. Genes that exhibited consistently significant differential expression (P < 0.05) and a uniform direction of change across all datasets were ultimately selected as final biomarkers.

### GSEA analysis of single genes

The “h.allmark.v7.4.symbols.gmt” gene set from the MSigDB database was chosen for more insights into molecular mechanisms of biomarkers in AMI. The R package clusterProfiler (Version 4.6.2), used pathway enrichment analysis for all samples in AMI training cohort based on Hallmark gene set of MSigDB.We fix the weighting index exponent equal to 1, remove gene sets whose sizes fall outside the range of 10–500, and adjust p-values to account for multiple testing using Benjamini-Hochberg (BH) procedures. For optimal speedup, we use fgsea in the pathway analysis. In our case, we obtain from this analysis pathways whose p-values are below 0.05. We define significantly enriched when their normalized enrichment scores satisfy |NES| > 1, which have been ranked by their enrichment scores (in descending order) to identify biologically meaningful functional pathways. Finally, five of the most significantly enriched pathways are picked out for visualization.

### Immune Infiltration Analysis

To investigate immune cell infiltration in acute myocardial infarction, the CIBERSORT algorithm (R packages e1071 v1.7-13 and preprocessCore v1.56.0) was applied to evaluate the composition and activity of immune cells in each sample from the AMI training cohort, comparing the AMI group with the control group [22]. Immune cell fractions were estimated in each sample from the training set, and Wilcoxon tests identified differentially abundant immune cells (P < 0.05) between AMI and control groups. Subsequently, Spearman correlation analysis was performed to assess the relationships between the biomarkers and these differential immune cells, with the results displayed in a heatmap.

### Construction of Molecular Regulatory Networks

Transcription factors (TFs) targeting the biomarkers were retrieved from the JASPAR 2024 database within the NetworkAnalyst platform [14]. Subsequently, miRNAs regulating the biomarkers were predicted using the TarBase 8.0 database. The lncRNAs targeting these miRNAs were then identified via the miRNet 2.0 database. Finally, an integrated lncRNA-miRNA-biomarker regulatory network was constructed and visualized using Cytoscape software.

### Single-cell analysis

Single cell RNA sequencing data were processed and analyzed through the package Seurat (version 5.0.1) [23] using initial quality control to filter out low quality cells (cells expressing less than 200 or more than 10, 000 genes, or have more than 10% mitochondrial gene content) and genes present in less than 10 cells. The data were normalized through the NormalizeData function. The top 2000 highly variable genes were identified (Find Variable Features) and scaled (Scale Data). Initial quality control involved filtering out low-quality cells (those expressing <200 or >10,000 genes, or with >10% mitochondrial gene content) and genes detected in fewer than 10 cells. The data were then normalized using the Normalize Data function. Principle component analysis (PCA) was performed and the relevant PCs were chosen for subsequent clustering based on JackStraw and Elbow plots. Batch effects were removed utilizing the Harmony algorithm. Cell clusters were determined using a shared nearest neighbor graph and the Louvain algorithm (resolution = 0.2), UMAP followed by non-linear dimensionality reduction to identify cluster-specific marker genes was performed using the Find All Markers function (log2FC > 0.25; adjusted p-value < 0.05). We also visualized the expression and cell type distribution of the discovered biomarkers across all annotated cell type in AMI using UMAP plots by cross-referencing the putative markers with databases like PanglaoDB and CellMarker 2.0.

## Results

### Differential Expression Analysis between AMI and Control Groups

**Fig 1:**
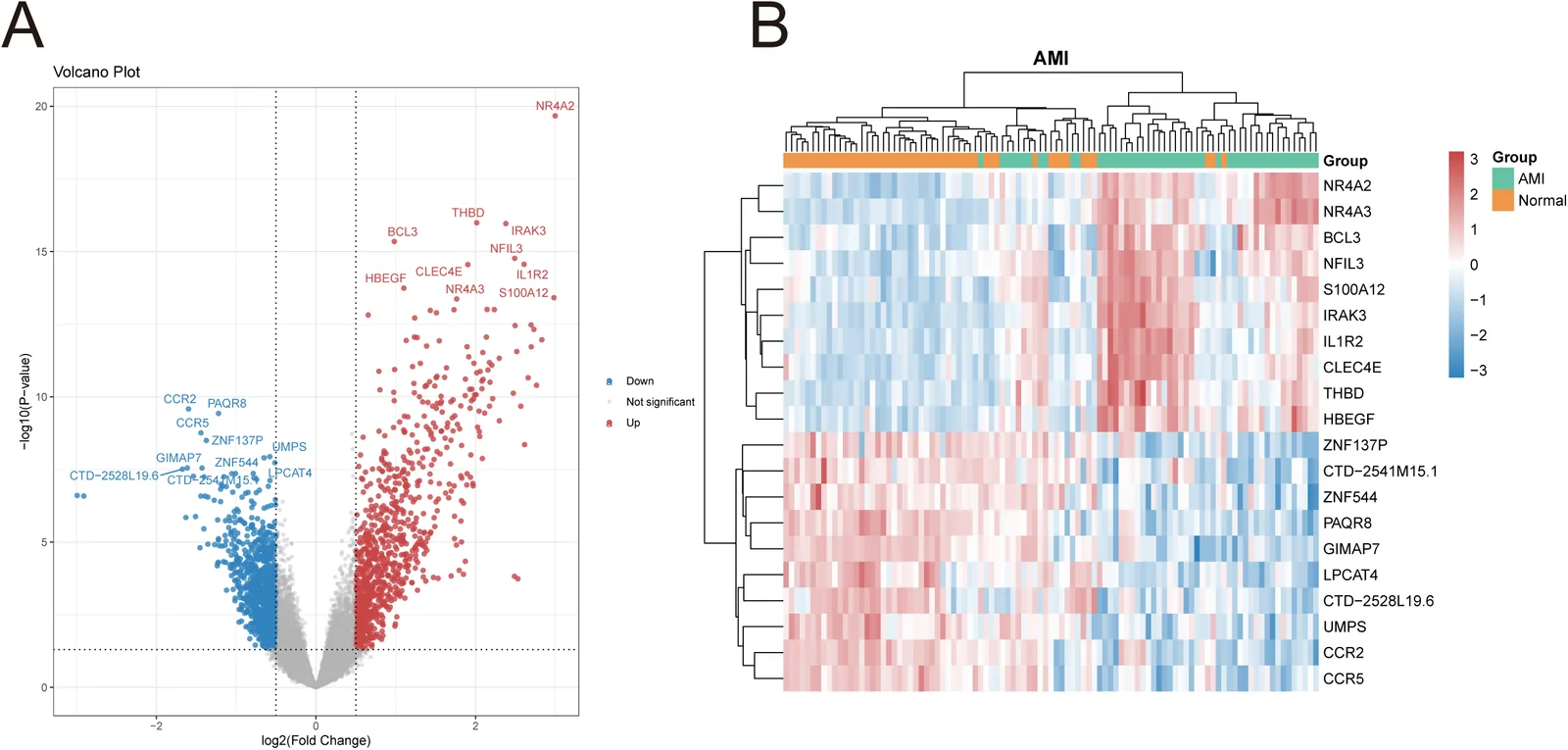
DEGs between AMI and control groups. (A) Volcano plot of DEGs in AMI and control groups. (B) Heatmap of the 20 DEGs in AMI and control groups.

Applying the pre-defined thresholds (P.Value < 0.05 and |log2FC| > 0.5), we identified a total of 1099 downregulated and 907 upregulated genes in the AMI training set (S2 and S3 Table). Volcano plots and heat maps were generated using the DEGs identified through screening to visualize the top 20 genes associated with AMI, as presented in Fig 1 A and B.

### WGCNA Identifies AMI-Associated Gene Modules

**Fig 2:**
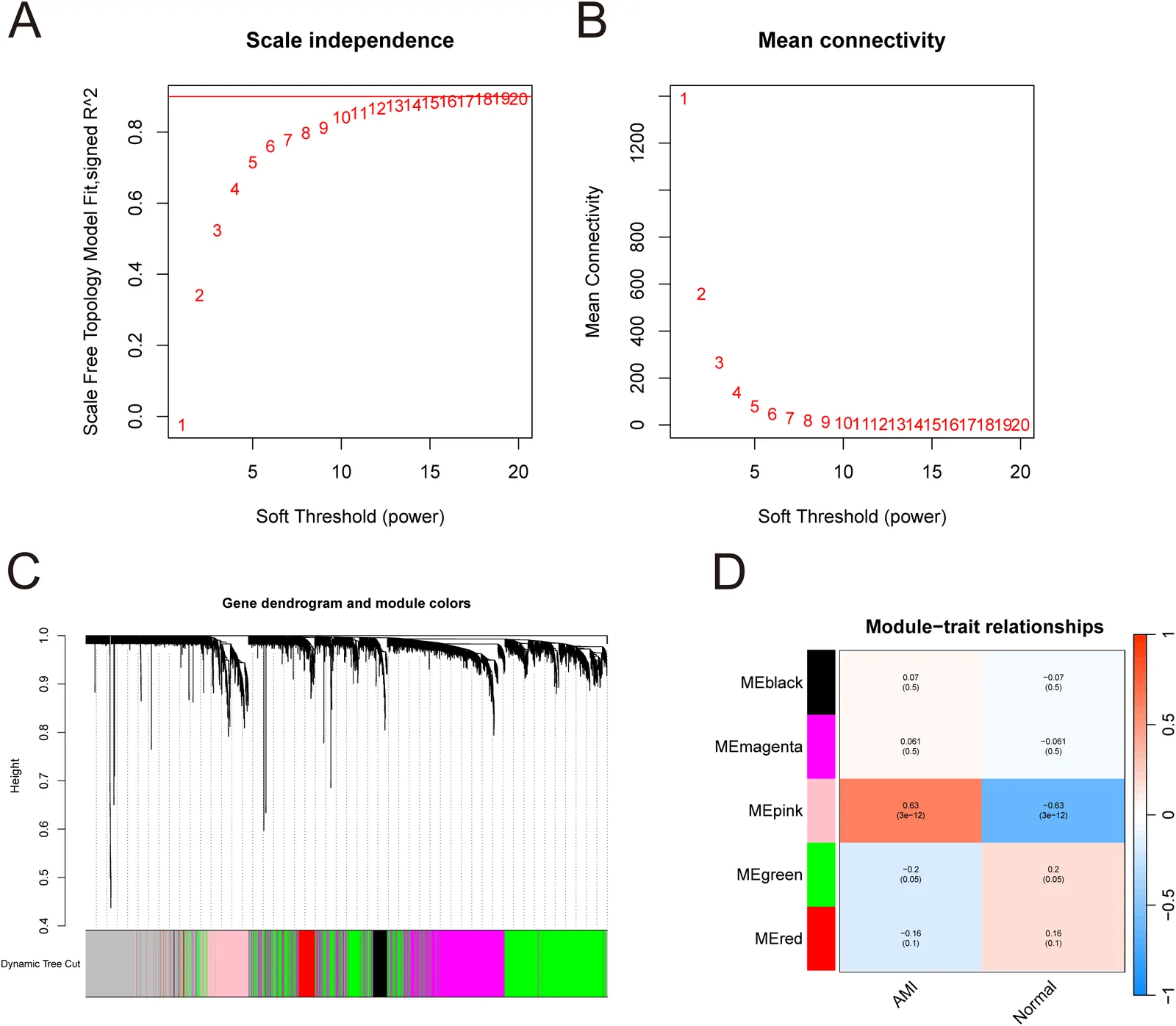
WGCNA of AMI. (A) Analysis of network topology for various soft-thresholding powers. (B) Mean connectivity plot corresponding to the soft-thresholding powers. (C) Cluster dendrogram of genes identified by WGCNA. (D) Module–trait relationships. Each cell contains the Pearson correlation coefficient and p-value.

Following the described methodology, a WGCNA was constructed from the AMI expression data. A soft thresholding power of 16 was selected to achieve a scale-free topology (Fig 2A, B). Highly correlated genes were then clustered into modules using dynamic tree cutting. These initial modules were subsequently merged if their correlation exceeded 0.75 (dissimilarity < 0.25). After merging and excluding the grey module, a final set of 5 distinct co-expression modules was defined (Fig 2C). The correlation between each module eigengene and the disease phenotype was calculated (S4 Table). As displayed in Fig 2D, the pink module demonstrated the most significant correlation with AMI (438 genes; correlation coefficient |r| = 0.63, p-value < 0.05). Consequently, the genes within the pink module were defined as the key AMI-related genes for subsequent analysis (S5 Table).

### Identification of candidate genes

**Fig 3:**
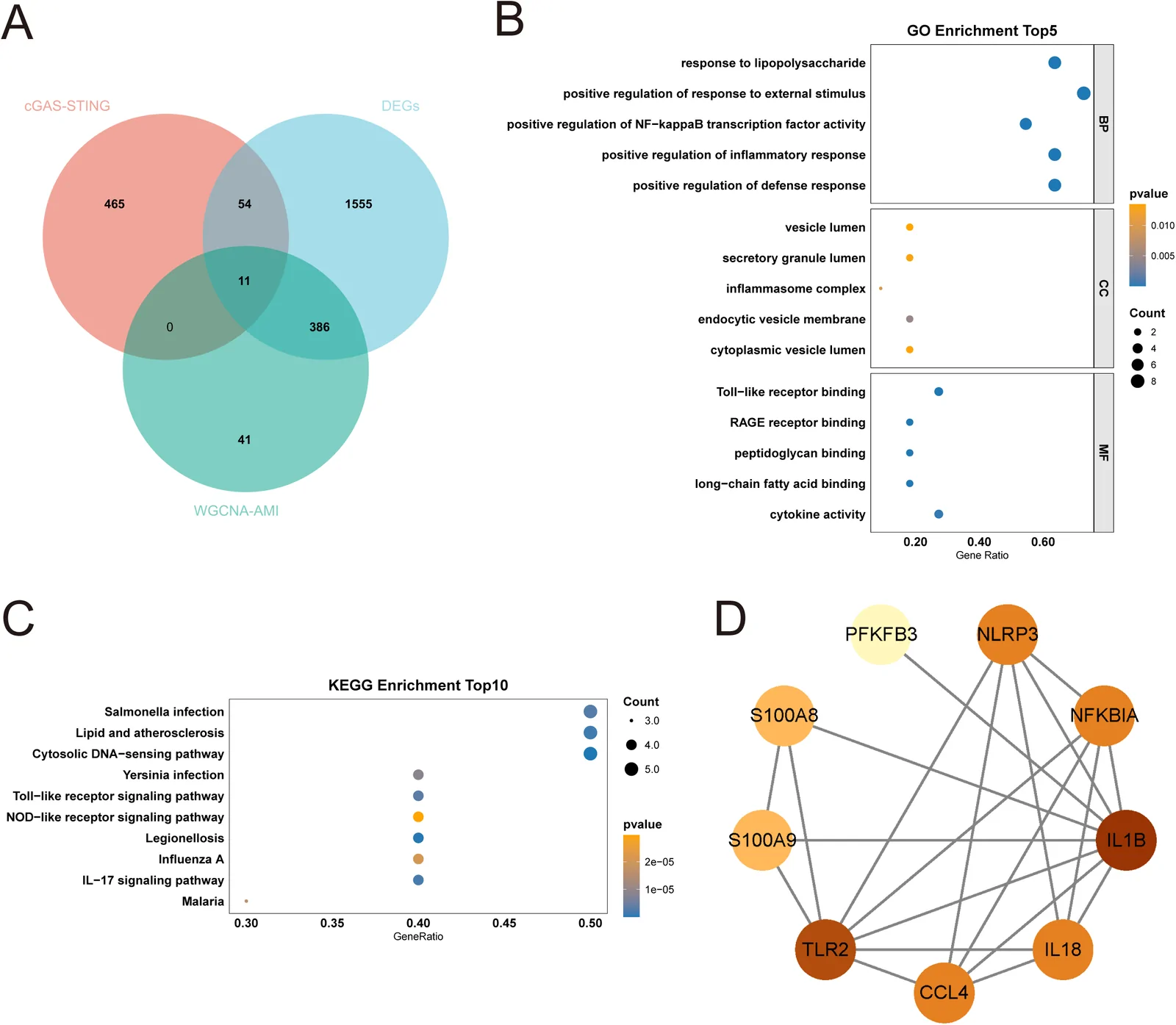
Identification of candidate genes. (A) Venn diagram of DEGs, co-espression modules, and cGAS-STING pathway-associated genes. (B) Top 5 GO enrichment analyses. (C) Top 10 KEGG enrichment analyses. (D) PPI network of the 11 candidate genes.

The Venn diagram of DEGs and WGCNA AMI-related genes and genes of pathway of cGAS-STING gave us 11 core candidate genes (Fig 3A and S6 Table). A functional annotation of 11 candidate genes conducted by GO and KEGG enrichment analyses (S7 and S8 Table). The given significance level was p-value < 0.05 and count > 1 and the significantly enriched GO terms were 294 with these criteria.The above contained 269 biological process (BP) terms mainly concerning the positive regulation of inflammatory response, defense response, and response to external stimulus; 6 cellular component (CC) terms, e.g., endocytic vesicle membrane and secretory granule lumen; and 19 molecular function (MF) terms, including Toll-like receptor binding, RAGE receptor binding and longchain fatty acid binding.KEGG pathway analyses indicated 40 significant enriched pathways such as the Cytosolic DNA-sensing pathway, Legionellosis, and Lipid and atherosclerosis pathway, suggesting that the candidate genes are primarily responsible for initiating innate immune and inflammatory response, pathogen recognition, cellular chemotaxis and migration, which are functionally associated with signaling pathways of NF-κB, Tolllike receptor, and IL-17 signaling.The candidate genes are found to coordinately regulate some key biological processes involved in the infection, immune-related disease, and inflammatory processes based on the result. The top 5 most significant GO terms in each type and top 10 most significant KEGG pathways visualized by bubble plots were shown in Fig 3B and Fig 3C respectively. A PPI network for all 11 candidate genes was build via STRING database with medium confidence (interaction score > 0.4).The resulting network showed that 9 of these 11 genes were part of a connected cluster. IL1B was the most connected among all. TLR2, CCL4, NFKBIA, IL18, NLRP3, S100A9, S100A8, PFKFB3 followed. The combined PPI network is shown in Fig 3D.

### Machine learning analyses converged on four hub genes for AMI

**Fig 4:**
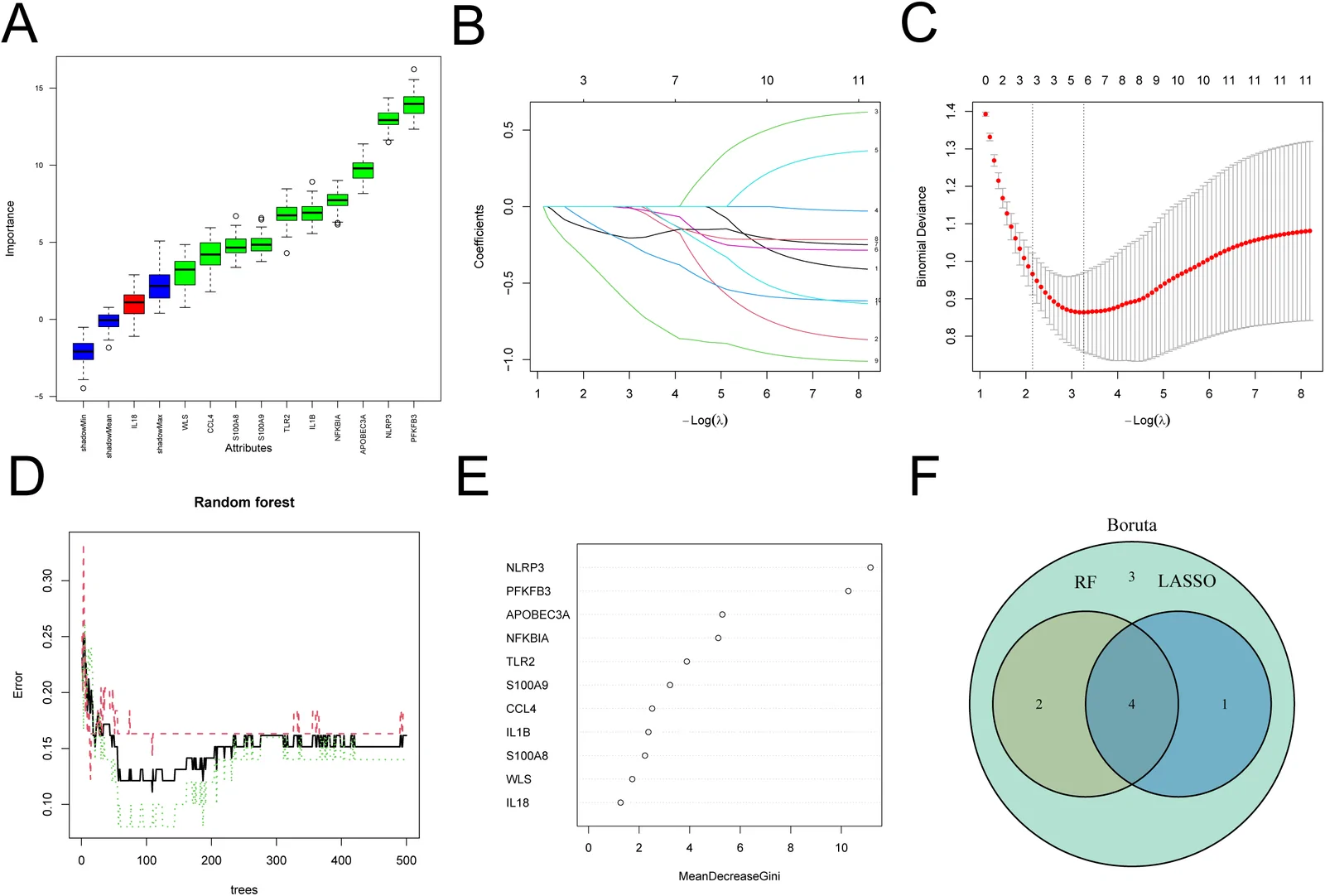
Identification of feature genes using machine learning algorithms. (A) Boruta algorithm for feature selection. (B) Feature selection via LASSO regression. (C) LASSO coefficient profile. (D) Error rates of the random forest model. (E) Forest plot of feature importance. (F) Four hub genes were identified through the intersection of Boruta, LASSO, and Random Forest.

The Boruta feature selection algorithm (Borzta and Kacprzyk, 2003), LASSO regression (Efron et al., 2004), and RF (Breiman, 2001) were used on the 11 candidate genes (for the AMI training set) as three different machine learning approaches to select stable feature genes. The number of relevant genes was found as 10 when the selected features compared to random shadow attributes with Boruta (Fig 4A). With LASSO regression, the features were selected using the addition of L1 regularization into the model and 5 genes were found (Fig 4B, C). At the same time, the RF algorithm picks up six genes according to the values of the features’ importance (Fig 4D, E). Lastly, by overlapping the gene sets generated by the three methods in the AMI training cohort, we find four genes in common as features: PFKFB3, TLR2, NLRP3, and APOBEC3A (Fig 4F and Table 1).

**Table 1:** Overlapping genes generated by the three methods in the AMI training cohort.

Boruta	LASSO	RF	Overlapping genes
NFKBIA	PFKFB3	NLRP3	PFKFB3
PFKFB3	TLR2	PFKFB3	TLR2
S100A8	IL1B	APOBEC3A	NLRP3
S100A9	NLRP3	NFKBIA	APOBEC3A
CCL4	APOBEC3A	TLR2	
TLR2		S100A9	
IL1B			
NLRP3			
APOBEC3A			
WLS			

### PFKFB3 andTLR2 are diagnostic biomarkers for AMI

**Fig 5:**
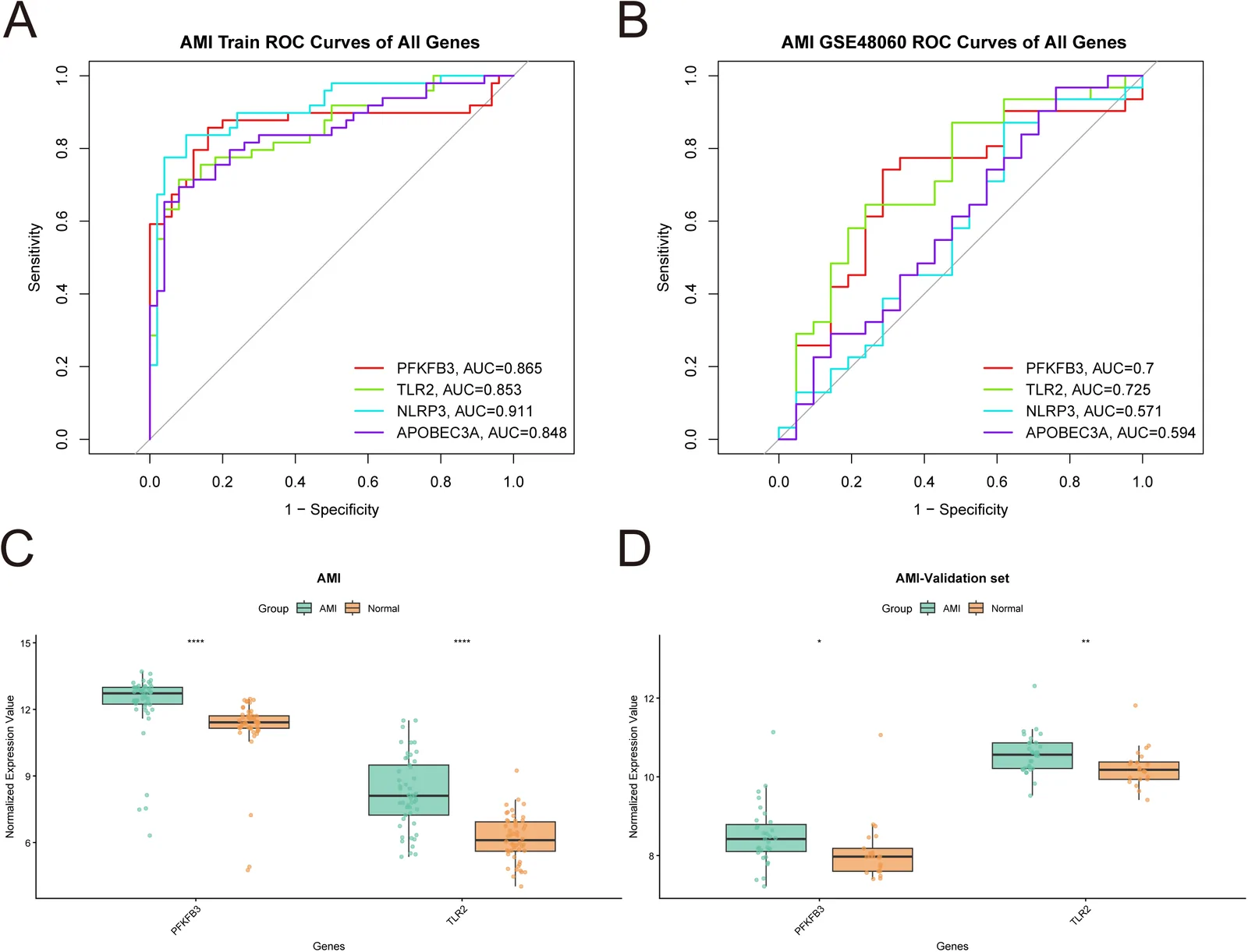
Evaluation of the identified biomarker in AMI. (A) ROC curve of the biomarker in the AMI training cohort. (B) ROC curve of the biomarker in the AMI independent validation cohort. (C) Box plot illustrating the expression levels of the biomarker in the training cohort. (D) Box plot illustrating the expression levels of the biomarker in the validation cohort *p < 0.05, **p < 0.01, ***p < 0.001, ****p < 0.0001.

The diagnostic performance of feature genes was measured by ROC curve in two training and validation AMI cohorts. AUC for all genes were above 0.7 (Fig 5A, B), from which two hub biomarkers PFKFB3 and TLR2 were chosen and validated for further analysis. In order to examine the generalizability of this selection of biomarkers, we did multi-dimensional validation in two independent datasets (GSE66360 as training set, GSE48060 as validation set).Differential expression analysis performed with the Wilcoxon test was also able to identify a differential expression of both PFKFB3 and TLR2, in both groups of AMI patient vs. control subjects (Fig 5C, D), with consistent identification of both PFKFB3 and TLR2 as useful diagnostic biomarkers of AMI.

### GSEA analysis of single genes

**Fig 6:**
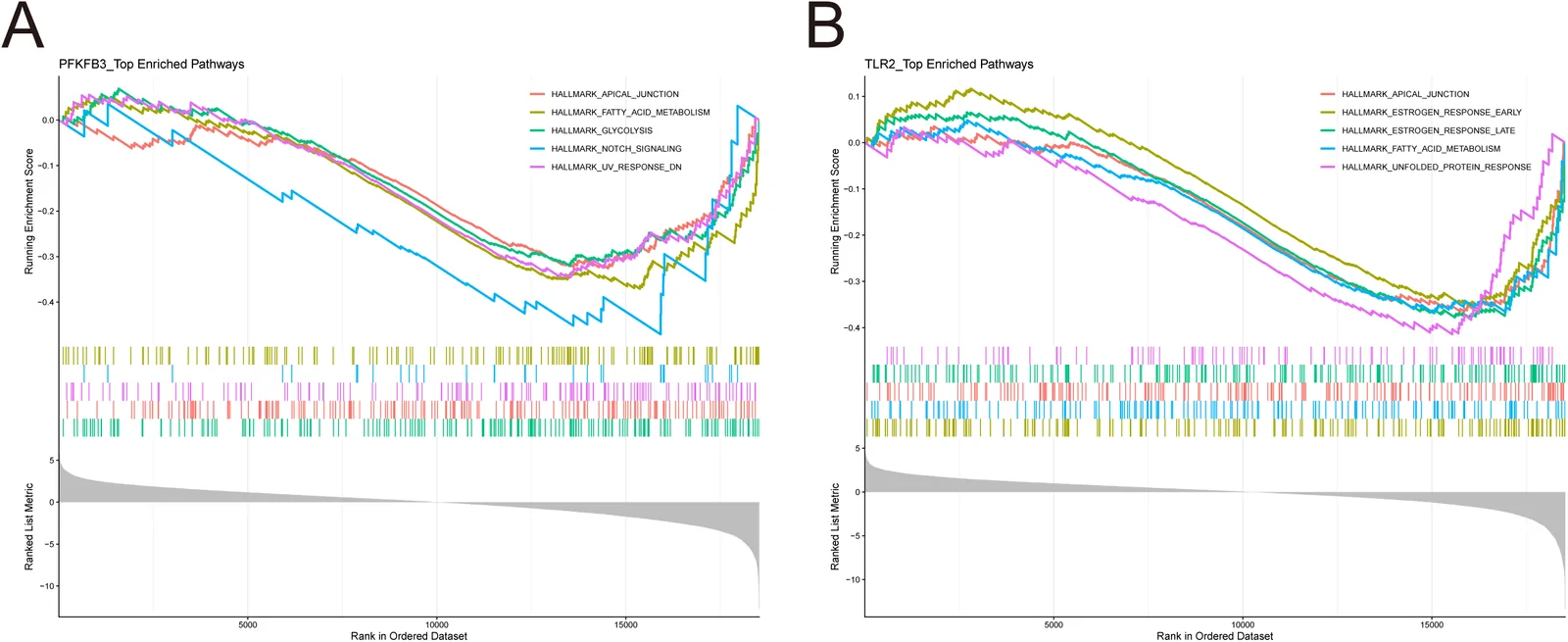
GSEA functional analysis of potential biomarkers. (A, PFKFB3; B, TLR2).

GSEA was conducted on the candidate biomarkers PFKFB3 and TLR2 by using the Hallmark gene sets from MSigDB. Enrichment significance was defined as an adjusted p-value (p. adjust) < 0.05 and | NES | > 1 (Fig 6A, B). In the AMI training set, PFKFB3 was significantly enriched in 40 Hallmark pathways such as the “GLYCOLYSIS”, “APICAL_JUNCTION” and “UV_RESPONSE_DN” (S9 Table).TLR2 was found enriched in 33 pathways, including “ESTROGEN_RESPONSE_EARLY”, “FATTY_ACID_METABOLISM” and “APICAL_JUNCTION” (S10 Table). The coordinated biological programs were identified as follow. HALLMARK_GLYCOLYSIS was significantly increased in this way while HALLMARK_HYPOXIA was heavily involved in upregulation as well. It might suggest critical energy metabolic reprogramming since the cancer cells were found to have hypoxic microenvironment. Co-activation of cell cycle machinery (“G2M_CHECKPOINT”, “E2F_TARGETS”, “MITOTIC_SPINDLE”) and increased DNA damage repair enzymes reflected a systemic attempt toward homeostasis of proliferative state. Of interest is the enrichment of more than one growth factor signaling pathways such as HALLMARK\_KRAS_SIGNALING_UP, HALLMARK\_TGF_BETA_SIGNALING, HALLMARK\_PI3K\_AKT\_MTOR_SIGNALING,HALLMARK\_MTORC1_SIGNALING, and their coordinated activity with the HALLMARK\_P53_PATHWAY, indicating the high involvement of signaling pathway in mediating the sub-underlying pathophysiological mechanisms. HALLMARK_INTERFERON_ALPHA_RESPONSE, HALLMARK_INTERFERONGAMMA_RESPONSE, HALLMARK_ALLOGRAFT_REJECTION, and HALLMARK_COMPLEMENT are upregulated, and HALLMARK_IL2_STAT5_SIGNALING is activated, which means that the body’s immune monitoring system is much more activated. Parallel up-regulation of pathways involved in modulating cellular architecture rearrangements (HALLMARK_APICAL_JUNCTION, HALLMARK_EPITHELIAL_MESENCHYMAL_TRANSITION) and secretory processes (HALLMARK_PROTEIN_SECRETION) suggest that the loss in barrier function may play a role on disease pathogenesis. In addition, there is extensive overlap between dysregulations in metabolic homeostasis networks (i.e., HALLMARK_FATTY_ACID_METABOLISM, HALLMARK_OXIDATIVE_PHOSPHORYLATION, HALLMARK_CHOLESTEROL_HOMEOSTASIS, HALLMARK_HEME_METABOLISM, and HALLMARK_XENOBIOTIC_METABOLISM), and stress responsive (e.g., HALLMARK_UNFOLDED_PROTEIN_RESPONSE, HALLMARK_UV_RESPONSE_DN, HALLMARK_UV_RESPONSE_UP, HALLMARK_TLR signaling. This is illustrated by HALLMARK_REACTIVE_OXYGEN_SPECIES_PATHWAY), which relates to metabolic regulation as an integral component of the underlying pathophysiology together with the stress response.

### Immune Cell Infiltration Analysis in AMI

**Fig 7:**
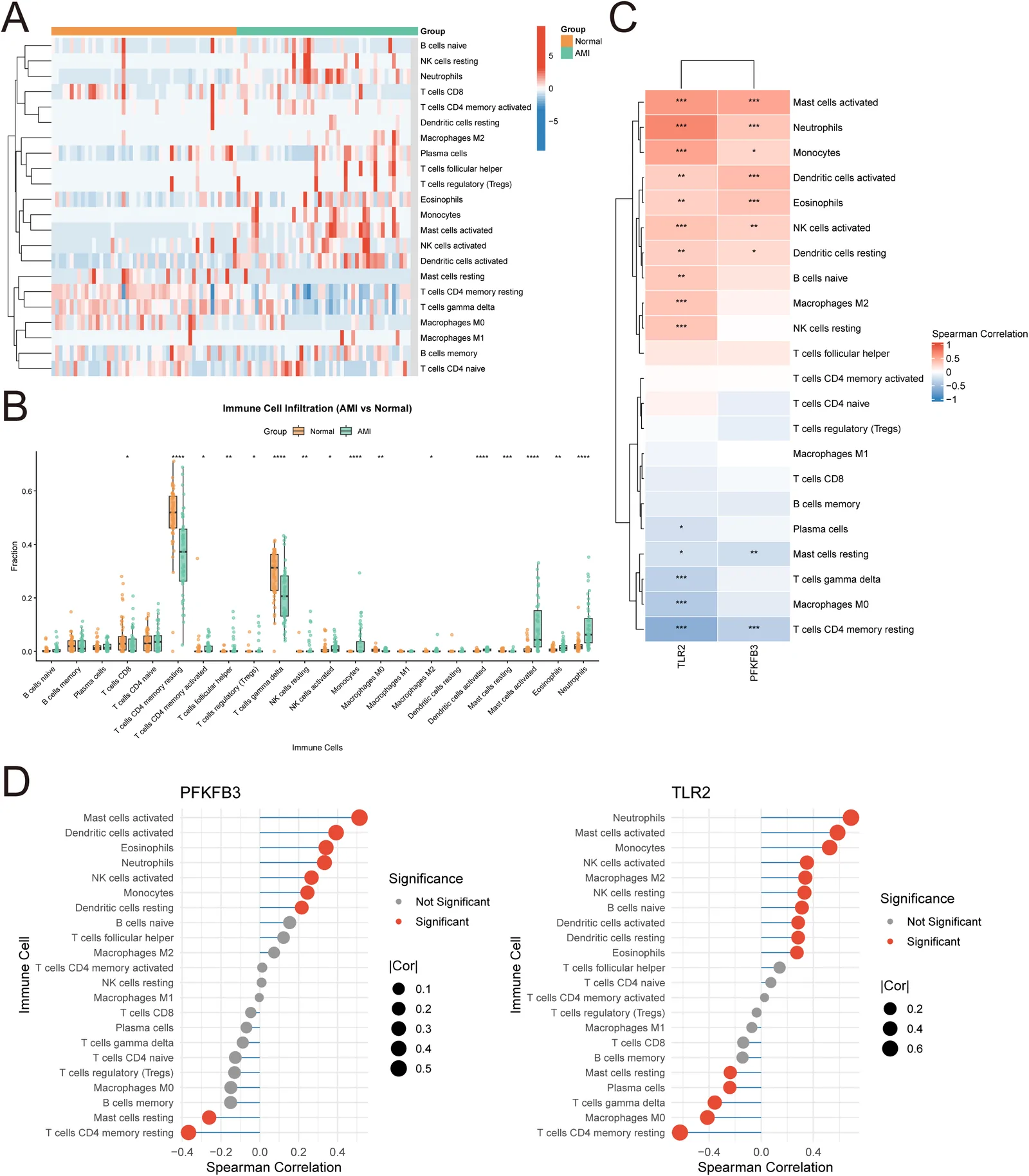
Exploring AMI-associated immune characteristics and the immune infiltrating cells associated with hub genes. (A) Heatmap of immune infiltration in AMI. (B) Box plot of immune infiltration in AMI. (C) Lollipop chart of the correlation between biomarkers and immune cells in AMI. (D) Heatmap of the correlation between biomarkers and immune cells in AMI. *p < 0.05, **p < 0.005, ***p < 0.001, ****p < 0.0001.

The immune cell infiltration profile between the patients with AMI and the healthy controls was compared and analyzed by CIBERSORT algorithm in this study (Fig 7A, B).Meaningful disparity was found between acute myocardial infarction and healthy controls in proportions of 16 immune cells subsets, among which CD8 + T cells, resting memory CD4 + T cells, activated memory CD4 + T cells, follicular helper T cells, regulatory T cells (Tregs), gamma delta T cells, rest NK cells, activated NK cells, rest and activated mast cells, eosinophils, neutrophils, monocytes, activated M1 macrophages, resting and activated M2 macrophages, activated dendritic cells. To further test the relationships between immune cell populations and biomarkers in the AMI training cohort, we used the psych R package, whose results are illustrated in Fig 7C and D. PFKFB3 and TLR2 were significantly positively correlated with activated NK cells, monocytes, resting and activated dendritic cells, activated mast cells, eosinophils and neutrophils in this AMI cohort, indicating potentially an aid role in activating and/or functional up-regulating immune cells.TLR2 was significantly positively associated with naive B cells, with resting NK cells and with M2 macrophages, which could also explain higher immune activity; while PFKFB3 as well as TLR2 was significantly negatively associated with resting memory CD4 + T cells and with resting mast cells, suggesting also a suppression effect on immune cell activity. Furthermore, TLR2 showed strong negative correlation with plasma cells and gamma delta T cells as well as M0 macrophages, which further suggested suppressive role for the host in some immune subgroups.

### Regulatory network analysis of candidate biomarkers

**Fig 8:**
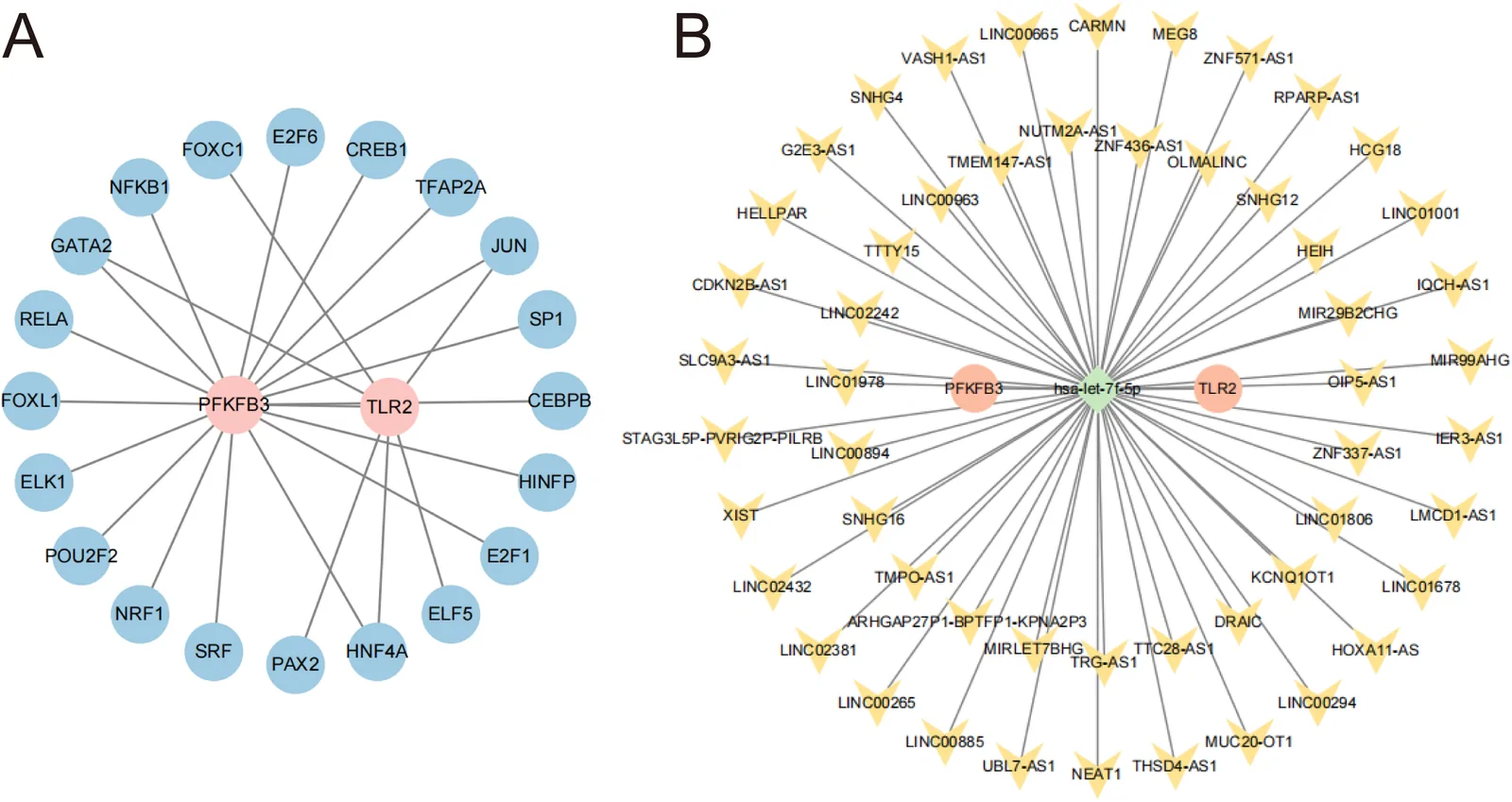
Molecular regulatory networks. (A) TFs regulatory network. (B) Competitive endogenous RNA (ceRNA) regulatory network (lncRNA-miRNA-biomarker). Biomarkers, pink; TFs, blue; miRNAs, green; lncRNAs, orange.

The regulatory network linking biomarkers and transcription factors (TFs) was visualized using Cytoscape (Fig 8A). A total of 20 TFs were identified as being associated with the biomarkers of interest. Specifically, PFKFB3 showed interactions with 18 TFs: JUN, GATA2, HNF4A, CEBPB, CREB1, FOXC1, POU2F2, SRF, E2F1, HINFP, SP1, TFAP2A, E2F6, NFKB1, RELA, ELK1, NRF1, and PAX2. TLR2 was linked to 7 TFs: FOXC1, JUN, FOXL1, PAX2, HNF4A, GATA2, and ELF5 (S11 Table). The lncRNA-miRNA-biomarker regulatory network was constructed and visualized using Cytoscape (Fig 8B). One miRNA, hsa-let-7f-5p, was identified to be associated with the biomarkers, while 53 lncRNAs—including ZNF436-AS1, SNHG12, MIR29B2CHG, LINC01806, LMCD1-AS1, MUC20-OT1, LINC00885, LINC02432, SLC9A3-AS1, and LINC02242, among others—were found to interact with this miRNA (S12 Table).

### PFKFB3 and TLR2 are Predominantly Expressed in Macrophages in AMI

**Fig 9:**
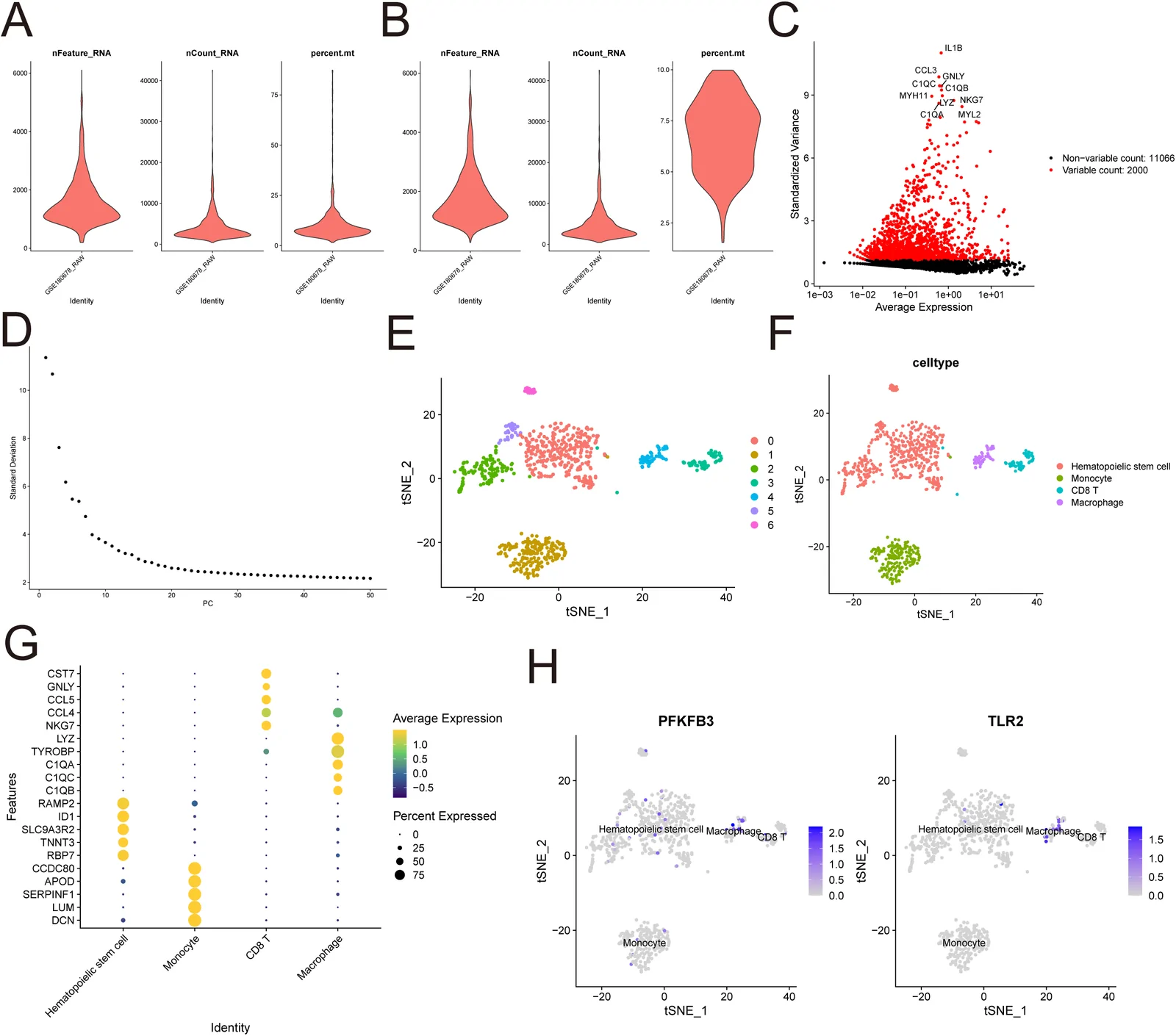
Single-cell RNA sequencing analysis. (A) Data before quality control. (B) Data after quality control. (C) Highly variable genes. (D) Elbow plot for dimensionality reduction. (E) UMAP visualization of unannotated cell clusters (0-6). (F) UMAP visualization of annotated cell types. (G) Expression of marker genes used for cell type annotation. (H) Distribution of biomarkers across the single-cell data.

Using the above approach, we applied a rigorous quality control for the AMI single-cell dataset. The cells were removed if they had less than 200 genes detected or greater than 10, 000 genes detected, or had a value greater than 10% ratio for mitochondrion genes or greater than 5% ratio for erythrocyte genes. We also removed genes detected in less than 10 cells (Fig 9A, B), which lead to a total of 794 high quality cells and 13 066 genes. Next, highly variable top 2,000 genes were determined for the downstream analysis (Fig 9C). Principal component analysis (PCA) was implemented and the first 30 principle components were chosen for further computational analysis, following the standard statistical criterion (Fig 9D). In order to have a characterization of the cell types within the single-cell dataset the cells were first visualized using t-SNE. Fig 9E presents the visualization of the unannotated sample, where seven clear clusters could be observed. After annotated the cells with known marker genes, we grouped the cells into 4 main population, i.e., Hematopoietic stem cell, Monocyte, CD8 T cell and Macrophage (Fig 9F) respectively. And we checked the marker gene expression pattern of these cells grouped to population, and visualized it as a dot plot (Fig 9G). We also checked the two biomarker PFKFB3, TLR2 expression pattern across cell populations within single cell dataset of AMI. They were both predominantly expressed in the macrophages, pointing to a possible functional relevance of these genes in this cell type (Fig 9H).

As shown in Fig 9, following the described method, we performed quality control on the single-cell data of acute myocardial infarction. The quality control conditions included low-quality cells with less than 200 genes and genes expressed in less than 10 cells, cells with less than 200 or more than 10,000 expressed genes, cells with a mitochondrial gene ratio greater than 10% and a red blood cell gene ratio greater than 5% (Fig 9A, B), and selected the top 2000 genes with the highest expression variability (Fig 9C). After quality control, 794 cells and 13,066 genes were obtained. The first 30 principal components were selected through principal component analysis for subsequent analysis (Fig 9D).

For figuring out the identity of cell from the single cell data, a single-cell t-SNE was first plotted. Fig 9E presented 7 clusters within the dataset before the cell type is known. After the cells are labeled according to defined marker gene expression, four major cell types including hematopoietic stem cells, monocytes, CD8 + T cell and macrophage are obtained (Fig 9F). We also visualized the expression patterns of the signature genes of each subpopulation across identified subpopulation of the clusters using bubble plot (Fig 9G). The full list for differently expressed genes for annotations is summarized in the S13 Table.

## Discussion

AMI is a serious and life-threatening cardiovascular pathology with a complex pathogenesis involving several biological pathways, including especially immune activation and the inflammatory processes. The pathogenesis of AMI is usually related to the poor blood flow of the coronary arteries leading to ischemic destruction of the myocardial cells. As global statistics show, acute myocardial infarction is one of the major causes of heart disease related death and weighs heavily mentally and economically on patients and their families. Despite multiple treatments existing currently, early diagnosis and effective treatment still face enormous problems. Therefore the profound studies of pathological mechanism of AMI and its related biomarkers have great importance in clinical aspects.

The present work is aimed at the comprehensively characterization of biomarkers based on cGAS-STING pathway in AMI and exploring the biological basis of these biomarkers. The identification of biomarkers (especially up-regulated genes) associated with AMI, by mining and analyzing the high throughput datasets using bioinformatics methods, so as to provide insights for diagnosis and therapy of AMI. Here, we are going to discuss on the results of studies and further look into the biological roles of DEGs and the contributions that are possibly involved in pathogenesis of AMI. Moreover, we are going to discuss diagnostic potency as well as a potential of the DEGs for personalized medicine in the early stages of disease diagnosis.

Herein, DEG analysis was applied on peripheral blood transcriptomic data from AMI patients. A WGCNA was then built to find gene modules well associated with the AMI phenotype. At the end, by considering DEGs, genes in modules highly associated with the AMI phenotype (after WGCNA) and genes related to the cGAS-STING pathway, we have found 11 candidate genes. In GO and KEGG Functional enrichment analyses, we found that these genes were mainly enriched in pathways involving the regulation of immune and inflammatory processes. As increasing evidence shows, after AMI, DAMPs are released from the necrotized cardiomyocytes and may activate the innate immune signaling to induce the downstream immune/inflammation cascade [24]. Also, KEGG pathway enrichment suggested that some candidate genes were involved in several known inflammatory signaling cascades such as NF-κB and IL-17, as well as cytokine-mediated networks. Immune cell infiltration further revealed that immune repertoire of AMI patient has changed significantly, consistent with previous findings immune activation in both animal models and clinical blood samples [25]. We note that, while inflammation and immune regulation are broadly believed to be key pathways in AMI, there remains discrepancies between papers in the relative importance of each individual pathway, and the importance of these pathways to repair of the damaged myocardium. For instance, some studies point out that apoptosis and autophagy would play a more definitive role in patient outcome and might even play a bigger role in deciding upon post-infarction prognosis [26]. Our high-dimensional data and multi-dimensional enrichment analysis conveys the dominance of immune-inflammatory pathway in AMI pathogenesis while acutely specifying the pathway affiliations and regulating networks of the candidate genes identified. Colchicine, a plant-based anti-inflammatory medication, has demonstrated efficacy in reducing the risk of major adverse cardiovascular events at low doses relative to placebo in clinical studies [24,27]. Despite these findings, they have not yet been integrated into current clinical guidelines, as additional research is required to confirm their clinical applicability [28]. Our study demonstrates that inflammation plays a key role in the progression of AMI which could support the potential use of colchicine as a therapeutic option for this condition.

The cGAS-STING pathway, serving as a central nucleic acid-sensing mechanism, plays a crucial role in mediating inflammatory signaling and facilitating immune cell infiltration in the context of cardiovascular disorders [9]. Existing studies suggest that the cGAS-STING pathway not only governs the synthesis of type I interferons but also enhances chemokine expression, thereby amplifying inflammatory reactions within the myocardium and facilitating systemic immune activation [29]. In mice with myocardial infarction, the cGAS-STING signaling pathway can be activated by interferon-inducible cells (IFNICs) derived from monocytes in the infarcted area, promoting the production of type I interferons and participating in ventricular remodeling after myocardial infarction [30]. However, knockout of cGAS, IRF3, and IFNAR genes or administration of type I interferon receptor neutralizing antibodies can effectively improve ventricular remodeling in mice [30]. Suppression of cGAS expression following AMI enhances the polarization of macrophages toward the M2 phenotype, which contributes to attenuating myocardial damage and facilitating cardiac repair in the post-infarction phase [31]. Thus, suppression of the cGAS-STING signaling pathway activation may offer potential therapeutic benefits in the treatment of myocardial infarction.

Our research found that PFKFB3 and TLR2 both demonstrated high AUC values (all greater than 0.7) in both the training set and the validation set, indicating a good ability to distinguish AMI patients from the control group. PFKFB3, a key glycolytic enzyme, is rapidly upregulated during myocardial ischemia and the repair process, thereby facilitating metabolic reprogramming and contributing to myocardial fibrosis development [32]. Inactivation of PFKFB3 protects the heart from myocardial fibrosis, left ventricular systolic dysfunction and dilation, as well as myocardial infarction injury induced by ligation of the left anterior descending coronary artery in mice, possibly by inhibiting the TGFβ-SMAD2/3 signaling pathway [32,33]. Recent evidence indicates that macrophage-specific suppression of PFKFB3 can mitigate inflammatory responses following ischemia/reperfusion injury by enhancing VEGF-C-dependent lymphangiogenesis [34]. An increasing amount of evidence indicates that PFKFB3-driven glycolysis has a pro-inflammatory effect in vascular endothelium. Mechanistically, enhanced glycolytic activity and inflammatory responses are driven by increased PFKFB3 expression; conversely, knocking down PFKFB3 genetically or inhibiting it pharmacologically dampens both glycolysis and inflammation, while forced PFKFB3 expression leads to their upregulation [35]. Moreover, PFKFB3 was identified as a novel transcriptional target of the NF-κB signaling pathway, previously uncharacterized in this context [35]. Inactivation of NF-κB signaling abolished the upregulation of both PFKFB3 expression and glycolytic activity; importantly, forced PFKFB3 expression in inflamed human PAECs1 fully reversed these deficits, providing strong evidence that the NF-κB–PFKFB3 axis coordinates immune-metabolic responses in vascular endothelial cells [35]. Other studies have shown that PFKFB3 can mutually regulate the activity of NF-κB, as inhibiting PFKFB3 would deactivate the NF-κB signal, thereby suppressing inflammation in VECs [36, 37]. Guo et al. further demonstrated that PFKFB3 activity is activated in an NADH-dependent manner via the C-terminal binding protein (CtBP), which regulates NLRP3 inflammasome activation by dissociating from its binding partner forkhead box P1 (FOXP1) [36]. The loss of PFKFB3 and the reduction of glycolytic activity decreased the number of macrophages in the plaque and impaired their cell proliferation function, leading to increased lesion instability and core necrosis [38].

Meanwhile, TLR2 acts as a pattern recognition receptor that recognizes both pathogen-associated molecular patterns and endogenous damage-associated molecular patterns (DAMPs), thereby triggering NF-κB signaling and the release of proinflammatory cytokines—key mechanisms closely associated with the inflammatory storm in the acute phase of AMI [39]. Research has found an atypical activation of systemic TLR response in patients with ST-segment elevation myocardial infarction (STEMI), characterized by increased transcriptional expression of TLR2 and TLR4 in circulating immune cells, accompanied by limited induction of MyD88, NF-κB and HIF-1α-dependent activation programs [40]. This response can be detected in the early stage of infarction, is associated with the ischemic period, and precedes the release of troponin T [40]. TLR and the subsequent activation of nuclear factor κB are key steps in the pathogenesis of I/R injury. Studies have shown that the lack of TLR2 signaling has a protective effect on myocardial cell and endothelial cell dysfunction [41]. In a porcine model, the use of clinical-grade humanized TLR2 antagonist OPN-305 to inhibit TLR2 before reperfusion reduced infarct size and improved systolic function [42].

In this study, by integrating multi-omics analysis with machine learning-based screening, we for the first time clearly identified PFKFB3 and TLR2 as cGAS-STING-associated diagnostic markers for AMI, thereby expanding the clinical utility of dual-pathway inflammation-metabolism biomarkers. This may provide an important basis for the diagnosis of early AMI with not yet elevated troponin, and the combination of high-sensitivity troponin may have a complementary and coordinated role in the diagnosis of AMI. Furthermore, whether their expression levels are related to infarct size and cardiac dysfunction needs to be further investigated in dedicated clinical cohorts. Overall, as novel biomarkers, PFKFB3 and TLR2 hold promise for advancing early, high-sensitivity detection and precise stratification of AMI patients.

### Limitations

Several limitations of this work are noted. First, this study is a retrospective, electronic database analysis, and its inherent biases (such as inconsistent data collection and lack of standardized follow-up) may affect the robustness of the results. Second, although we used high-throughput RNA sequencing, the overall sample size was moderate, and no independent prospective validation cohort was included, which may statistically limit the full demonstration of differences between different subtypes of AMI. Therefore, the wide applicability and reproducibility of the identified candidate biomarkers (PFKFB3 and TLR2) still need further validation in larger-scale, independent cohorts. Third, this study relied on peripheral blood transcriptome data rather than expression profiles directly from cardiac tissue. Given that the core pathological process of AMI occurs in the myocardial microenvironment, the blood transcriptome may not fully reflect the local changes in the heart, especially those related to the cGAS-STING pathway. Fourth, this study lacks direct experimental validation of the cGAS-STING pathway (such as gene knockout, overexpression, or drug intervention), so the proposed mechanism by which PFKFB3 and TLR2 regulate the pathophysiology of AMI through this pathway remains a bioinformatics speculation and needs to be confirmed by functional experiments. Fifth, the single-cell analysis results involved in this study are only of preliminary exploratory nature and have not been verified through wet experiments or independent single-cell datasets. The cell type specificity and dynamic expression patterns still need further investigation. Additionally, the technical heterogeneity between different datasets (such as experimental conditions and sequencing platform differences) may introduce potential batch effects, which may affect the stability of the overall results and the interpretability of gene expression changes.

## Conclusion

In conclusion, this study successfully identified PFKFB3 and TLR2 as key candidate biomarkers for AMI and preliminarily analyzed the possible cGAS-STING pathway mechanism they might be involved in based on bioinformatics methods, providing a new perspective for the early diagnosis and targeted treatment of AMI. However, it must be clearly pointed out that the above findings are still in the exploratory stage, and the identified biomarkers need to be strictly externally validated in larger-scale, multi-center prospective cohorts to assess their clinical applicability. At the same time, it is urgently necessary to confirm through in vitro and in vivo functional experiments (such as regulating the expression of PFKFB3/TLR2 in cardiomyocytes or animal models and detecting the activity of the cGAS-STING pathway) whether these biomarkers are truly involved in the pathophysiological process of AMI through the cGAS-STING axis. Future research should also further explore the interaction between the cGAS-STING pathway and other inflammatory signaling pathways to more comprehensively reveal the complex molecular network of AMI. Only after completing the above validation and functional analysis can the clinical translational value of PFKFB3 and TLR2 as AMI diagnostic markers or therapeutic targets be reliably evaluated.

## Supporting information

S1 Table Genes associated with the cGAS-STING pathway. Union of genes retrieved from PathCards (103 genes), MsigDB (18 genes), and GeneCards (466 genes).
(TXT)

S2 Table Downregulated genes in the AMI training set. 1,099 genes that were significantly downregulated in the AMI training set.
(TXT)

S3 Table Upregulated genes in the AMI training set. 907 genes that were significantly upregulated in the AMI training set.
(TXT)

S4 Table Correlations between module eigengenes and disease phenotype. This table lists the correlation coefficients and corresponding p-values between each module eigengene and the AMI disease phenotype in the training set.
(XLS)

S5 Table Genes in the pink module. This table lists the genes comprising the pink module, which were defined as the key AMI-related genes for subsequent analyses.
(TXT)

S6 Table 11 core candidate genes. 11 genes obtained from the intersection of DEGs, WGCNA AMI-related genes, and cGAS-STING pathway genes.
(TXT)

S7 Table GO enrichment analysis results. GO enrichment results for the 11 core candidate genes.
(TXT)

S8 Table KEGG enrichment analysis results. KEGG pathway enrichment results for the 11 core candidate genes.
(TXT)

S9 Table Hallmark pathways enriched for PFKFB3. 40 Hallmark pathways significantly enriched for PFKFB3 in the AMI training set.
(CSV)

S10 Table Hallmark pathways enriched for TLR2. 33 Hallmark pathways significantly enriched for TLR2 in the AMI training set.
(CSV)

S11 Table Transcription factors interacting with PFKFB3 and TLR2. This table lists the transcription factors that interact with PFKFB3 (18 TFs) and TLR2 (7 TFs).
(TXT)

S12 Table miRNA and interacting lncRNAs. This table lists the miRNA (hsa-let-7f-5p) associated with the biomarkers and the 53 lncRNAs that interact with it.
(TXT)

S13 Table Differentially expressed genes for cell type annotation. DEGs used to annotate cell types in the single-cell dataset.
(CSV)

## Abbreviations

AMI: acute myocardial infarction
DEGs: differentially expressed genes
WGCNA: weighted gene co-expression network analysis
cGAS-STING pathway: cyclic GMP-AMP synthase–stimulator of interferon genes pathway
RNA: Ribonucleic Acid; AUC: area under the curve
PFKFB3: fructose-2,6-biphosphatase 3
TLR2: Toll like receptor 2
DNA: Deoxyribonucleic Acid
CVDs: cardiovascular diseases
TMO: topological overlap matrix
KEGG: Kyoto Encyclopedia of Genes and Genomes
GO: Gene Ontology
PPI: Protein-Protein Interaction
LASSO: Least Absolute Shrinkage and Selector Operation
RF: Random Forest
ROC: receiver operating characteristic
BH: Benjamini-Hochberg
TFs: Transcription factors
PCA: Principle component analysis
UMAP: Uniform Manifold Approximation and Projection
BP: biological process
CC: cellular component
MF: molecular function
DAMPs: damage-associated molecular patterns
NF-κB: nuclear factor kappa-light-chain-enhancer of activated B cells
IFNICs: interferon-inducible cells
IRF3: Interferon Regulatory Factor 3
IFNAR: Interferon Alpha/Beta Receptor
TGFβ-SMAD2/3 signaling pathway: Transforming Growth Factor Beta-Small Mother Against Decapentaplegic 2/3 Signaling Pathway
VEGF-C-dependent lymphangiogenesis: Vascular Endothelial Growth Factor C-dependent Lymphangiogenesis
PAECs1: Pulmonary Artery Endothelial Cells
VECs: Vascular Endothelial Cells
CtBP: C-terminal binding protein
FOXP1: forkhead box P1
STEMI: ST-segment elevation myocardial infarction
HIF-1α: Hypoxia-Inducible Factor 1-alpha
